# Complexes of allergen and specific IgG antibodies activate human neutrophils

**DOI:** 10.3389/falgy.2026.1773565

**Published:** 2026-03-23

**Authors:** Bibiana Aigner, Gordana Wozniak-Knopp, Nahla Ibrahim, Ute Vollmann, Christine Brostjan, Barbara Bohle

**Affiliations:** 1 Institute of Pathophysiology and Allergy Research, Center for Pathophysiology, Infectiology and Immunology, Medical University of Vienna, Vienna, Austria; 2 Institute of Molecular Biotechnology, Department of Biotechnology and Food Sciences, BOKU University, Vienna, Austria; 3 Division of Vascular Surgery, Department of General Surgery, Medical University of Vienna and University Hospital Vienna, Vienna, Austria

**Keywords:** allergen, antigen—antibody complex, antigen-presentation, neutrophil, T cell response

## Abstract

**Aim:**

To investigate whether AICs activate neutrophils via Fc*γ*R and influence allergen-uptake and presentation

**Methods:**

Major birch pollen allergen was incubated with different monoclonal antibodies (mAbs) and their effector-attenuated LALA-variants. AIC formation was analysed by dynamic light scattering. Neutrophils isolated from birch pollen allergic and non-allergic donors were stimulated with GM-CSF and IFN-*γ*. Fc*γ*R expression and internalisation of fluorescence-labelled allergen were determined by flow cytometry. Neutrophils pulsed with allergen, AICs or LALA-AICs were subjected to DNA-release assays and served as APC for autologous allergen-specific T-cells.

**Results:**

Cytokine-primed neutrophils showed upregulated Fc*γ*RI/CD64, downregulated Fc*γ*RIII/CD16, and unaltered Fc*γ*RII/CD32 expression. AICs with one and two mAbs were generated. Neutrophils phagocytosed larger AICs and LALA-AICs more effectively than smaller complexes and non-complexed allergen. Compared to non-complexed allergen, T-cell proliferation was inconclusive when neutrophils were pulsed with AICs and enhanced when pulsed with LALA-AICs. AICs but not LALA-AICs induced NET-release.

**Conclusion:**

Fc*γ*R-independent phagocytosis of AICs enhanced the allergen-presenting activity of neutrophils but Fc*γ*R-mediated NET induction might interfere with the T cell stimulatory properties. Our results suggest a novel link between humoral and cellular responses to allergens.

## Introduction

Neutrophils comprise 50%–70% of all leukocytes circulating in the blood. They rapidly migrate to inflamed tissues where they perform different functions, e.g., phagocytosis, degranulation of proteinases, production of reactive oxygen species (ROS) and neutrophil extracellular traps (NETs) (1). Neutrophils can bind IgG by receptors for the constant (Fc) region, termed Fc gamma receptors (Fc*γ*R) (2). Thereby, antibodies equip neutrophils with antigen-specificity. Resting human neutrophils express mainly Fc*γ*RIIa (CD32a) and Fc*γ*RIIIb (CD16b) (3, 4). These activating low-affinity receptors bind IgG when multivalent in immune complexes (IC) (5, 6) and their aggregation results in different responses: CD32a triggers phagocytosis while CD16b induces nuclear factor activation and NET formation (2). Following activation with granulocyte-macrophage colony-stimulating factor (GM-CSF) and interferon (IFN)-*γ* neutrophils also upregulate the high-affinity Fc*γ*RI (CD64) which allows them to bind monomeric IgG1, IgG3, and IgG4, and to phagocytose IgG-opsonized pathogens (7, 8). In addition, neutrophils express the IgA-binding Fc*α*RI (CD89) (9, 10) and all three defined types of IgE receptors (Fc*ε*RI, Fc*ε*RII/CD23 and galectin-3) (11).

Inflammatory cytokines, and GM-CSF and IFN-*γ* in particular, were shown to prolong the lifespan of neutrophils and together with interleukin (IL)-3 promote a portion of them to express HLA-DR (12–14). Furthermore, the cytokines prompted neutrophils to internalise and rapidly process allergens and present them in MHC class II/peptide complexes on their surface (15). This signal 1 together with CD58 as costimulatory signal 2 activated allergen-specific CD4^+^ T cells to proliferate and to release cytokines (16). In turn, T cell-derived cytokines enhanced the antigen-presenting activity of neutrophils (13, 14). Together, these findings suggested that neutrophils can serve as antigen-presenting cells (APC) for allergen-specific effector T cells at sites of inflammation (17). Indeed, we detected HLA-DR-positive neutrophils in suction blisters from allergen-induced cutaneous late phase responses of allergic patients (15).

Repeated exposure to high doses of allergens induces the production of high levels of allergen-specific IgG, as reported for bee keepers or cat owners (18–20). In addition, patients undergoing allergen immunotherapy (AIT) produce high levels of polyclonal allergen-specific IgG antibodies. The latter mainly comprise IgG1 antibodies in the early phase of AIT which are later outnumbered by IgG4 (21, 22). Both isotypes can form immune complexes with allergens (AIC), some of which prevent the binding of IgE and subsequent induction of allergic symptoms (23–26). Here, we sought to study whether the encounter of AICs influences allergen-presentation of neutrophils. We employed the major birch pollen allergen, Bet v 1, as model allergen, three monoclonal IgG1 antibodies (mAb), and Bet v 1-specific T cells expanded from individuals with birch pollen allergy. AICs were added to cytokine-primed neutrophils and allergen uptake and T cell proliferation were compared to non-complexed allergen. To address the involvement of Fc*γ*R we included genetically modified LALA variants of the mAbs that carry leucine to alanine substitutions at positions 234 and 235 in the Fc region which drastically reduces the binding to Fc*γ*R (27).

## Material and methods

### Participants

Individuals with birch pollen allergy showed typical case histories (rhinoconjunctivitis in spring) and birch pollen-specific IgE levels of >0.35 kU_A_/L (ImmunoCAP; Thermo Fisher Scientific, Uppsala, Sweden). Non-allergic individuals displayed neither symptoms nor specific IgE. All individuals provided written informed consent. The study was approved by the ethics committee of the Medical University of Vienna (EK-1488/2017) and conducted in accordance with the Declaration of Helsinki.

### Isolation of neutrophils

Neutrophils were isolated as previously described (15). Briefly, heparinized blood was separated by centrifugation on Ficoll-Paque PLUS density gradient medium (Cytiva, Marlborough, Massachusetts, USA), followed by dextran sedimentation (4% Dextran 500, Carl Roth, Karlsruhe, Germany), and red cell osmotic lysis of the granulocyte pellet. This resulted in repeatedly >91% pure CD16^+^CD66b^+^CCR3^−^HLA-DR^−^CD19^−^CD3^−^CD14^−^ cells as determined by flow cytometry using the FACSCanto II with FACSDiva (BD Biosciences, San Jose, Calif) and FlowJo (TreeStar, Ashland, Ore) software. The following anti-human antibodies were used: fluorescein isothiocyanate-labeled CD14, CD32A/B (clone FUN-2) and HLA-DR, PerCP-labeled CD16 and CD64, CCR3-allophycocyanin, phycoerythrin-labeled CD16 (PE; clone 3G8) and CD32B/C (clone S18005H), and Brilliant Violet 510-labelled CD16 (all from BioLegend, San Diego, Calif), fluorescein isothiocyanate-labeled CD3 and CD19, and CD66b-PerCP (all from BD Biosciences, San Jose, Calif). Prior to the use of neutrophils as APC an immunomagnetic negative selection step using the human MACSxpress Neutrophil Isolation Kit (Miltenyi Biotec, Bergisch Gladbach, Germany) was carried out which resulted repeatedly in >99% neutrophils. Neutrophils were cultivated in RPMI 1640 supplemented with gentamicin (100 mg/L, both from Sigma-Aldrich, Darmstadt, Germany), glutamine (2 mM, Thermo Fisher Scientific Inc., Waltham, MA, USA), and 10% autologous plasma.

### Allergen and monoclonal antibodies

Recombinant Bet v 1.0101 (rBet v 1) was produced and characterized in house (26) and conjugated to pHrodo-succinimidyl ester (Invitrogen, Carlsbad, Calif), according to the manufacturer's instructions.

The sequences of mouse hybridoma BIP1 antibody (mAb1) variable domains as described in Laffer et al. (28) were ordered from Genscript. mAb2 and mAb3 variable chain sequences were isolated from Fab yeast display libraries based on peripheral blood mononuclear cells (PBMC) from two individuals after sublingual administration of rBet v 1 (29) as described (30), with sorting performed with biotinylated rBet v 1. Antibodies were cloned with human constant IgG1 heavy chain and kappa domains into pTT-based vectors (Canadian National Research Council). Effector-attenuated variants were obtained by mutagenesis of L234 and L235 (EU numbering) (31) to alanine residues. The expression proceeded in HEK293-6E cells for 5 days after polyethylenimine-mediated transfection, with the addition of tryptone TN-1 supplement to the end concentration of 0.5%. After harvest, the supernatant was filtered and buffered with 0.1 M Na-phosphate, pH 7, and purified using Protein A affinity chromatography, with elution in 0.1 M glycine, pH 3.5. Neutralized antibody-containing fractions were buffer-exchanged to PBS and the monomeric profile of products was monitored using size-exclusion chromatography in native conditions, on Superdex 200/GL run in PBS with 200 mM NaCl.

To form allergen-antibody complexes rBet v 1 or pHrodo-labelled rBet v 1 were incubated with equimolar amounts of mAbs or their LALA variants for 30 min (min) at room temperature.

### Dynamic light scattering (DLS)

DLS measures the time-dependent fluctuation of the light scattered by molecules in a solution to determine the translational diffusion coefficient, the hydrodynamic radius from the Stokes–Einstein equation (32). The primary result is the intensity-weighted distribution of the particles hydrodynamic diameter. The latter refers to individually moving entities, that is, single particles and particle aggregates. It typically reflects the outer dimensions, but it is virtually not related to the size of constituent particles within an aggregate. Measurements were performed using a Dynapro Nanostar DLS instrument (Wyatt Technology, Santa Barbara, MI), operating at *λ* = 658 nm, produced by a 120 mW air launched laser at a scattering angle of 90° at 25.0 ± 0.1 °C, using disposable cyclic olefin copolymer cuvettes (WYATT Technology, Santa Barbara, MI). The results are presented as individual graphs of dependency of scattered light intensity and particles size. Mean values of 20 acquisitions were evaluated.

### Allergen uptake

Flow cytometry was done after keeping neutrophils (2 × 10^6^×mL^−1^) without and with pHrodo-labeled rBet v 1 (2 µg/mL) or adjusted amounts of AICs and LALA-AICs in presence of GM-CSF (100 pg/mL), IFN-*γ* (10 ng/mL), and IL-3 (30 ng/mL; all from PeproTech, Rocky Hill, NJ) for the indicated time periods.

### T cell proliferation assay

Allergen-specific T cell cultures were expanded from PBMC of birch pollen-allergic patients as described (33). T cells (2–5 × 10^4^) were co-cultured with >99% pure autologous neutrophils (5 × 10^4^) that had been washed once after cultivation for 24 h (h) in GM-CSF/IFN-*γ*/IL-3 in the absence or presence of rBet v 1 (5 μg/mL) or adjusted amounts of AICs or LALA-AICs. All experiments were done in duplicates. After 48 h ^3^[H]-labelled thymidine was added for additional 16 h. Thymidine incorporation was assessed as counts per min (cpm). Delta cpm (dpm) = cpm of allergen-stimulated T cells minus cpm of unstimulated T cells were calculated. Cpm in T cell cultures without allergen ranged from 211 to 501 and with allergen from 782 to 1202 cpm. To be considered allergen-specific the cpm in response to allergen had to be more than twice as high as the cpm in medium controls.

### Neutrophil extracellular traps release assay

Following the established protocol (34) neutrophils (1 × 10^5^) were stimulated with the calcium ionophore A23187 (2.5 µM, Sigma-Aldrich, St. Louis, MO, USA) as commonly used positive control), rBet v 1 (2 µg/mL) or adjusted amounts of AIC1 and LALA-AIC1, or rBet v 1 (10 µg/mL) and adjusted amounts of AIC2, AIC3, and their LALA variants. These concentrations were determined in preliminary experiments. To quantify the DNA release, 5 μM of SYTOX Green (Thermo Fisher Scientific Inc.) was added and fluorescence was measured at 15 min intervals at excitation/emission of 485/520 nm using the VarioSkan Lux plate reader (Thermo Fisher Scientific Inc). All experiments were done in triplicates and mean values were calculated, so that each donor contributed a single value per condition and time point. Time course data are shown as the median relative fluorescence units (RFU) across donors at each time point. Where indicated, RFU values at 300 min are shown as median with individual points representing donors.

For visualization of NETs, immunofluorescence staining was performed as described (34). Neutrophils (2 × 10^5^) were incubated with the indicated stimuli for 180 min at 37 °C to allow NET formation. NETs were identified by staining for citrullinated histone H4 (CitH4, 1:500, 07–596, Merck, Darmstadt, Germany), a NET-associated marker released during NET formation, detected with an Alexa Fluor Plus 555 secondary antibody (1:400, Thermo Fisher Scientific Inc.). Released DNA strands were visualized using Hoechst 33342 (1:1,000, Invitrogen). Fluorescence images were acquired using an Axio Observer Z1 microscope (Carl Zeiss MicroImaging, Inc.) with a 20x objective and TissueFAXS scan software (TissueGnostics GmbH, Vienna, Austria).

### Statistics

Data were analysed by using GraphPad Prism 10.0 (GraphPad Software, Boston, MA) and IBM SPSS 28.0 software (SPSS, Chicago, Ill). Pairwise comparisons were performed using the Wilcoxon signed-rank test. Differences were considered statistically significant at a *P* value < 0.05.

## Results

### Cytokine-induced Fc*γ*R surface expression of neutrophils

**Figure 1:**
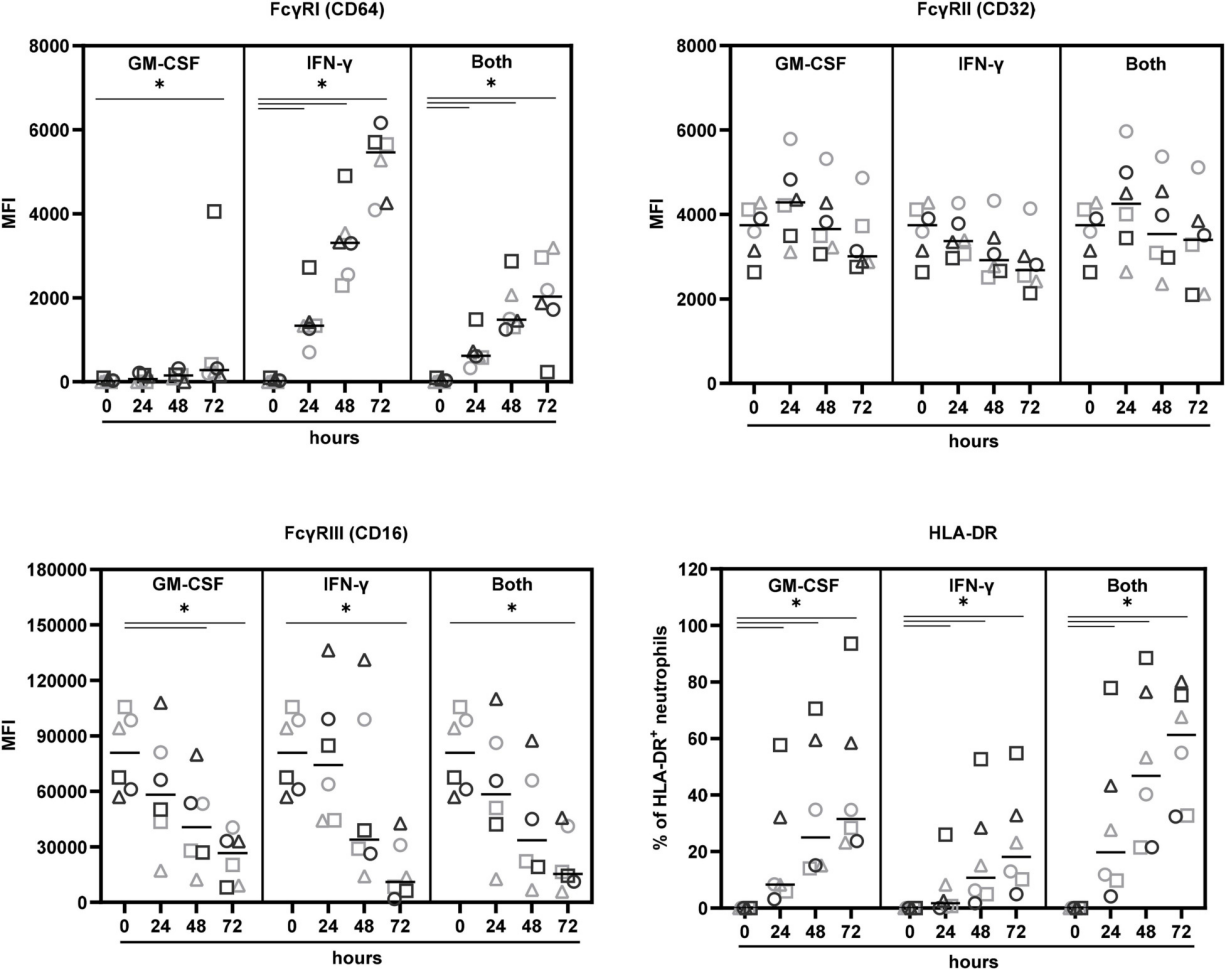
Expression of Fc*γ*R and HLA-DR on neutrophils. CD16^+^CD66b^+^CCR3^−^HLA-DR^−^CD19^−^CD3^−^CD14^−^ cells from 3 non-allergic (bright grey symbols) and 3 allergic (dark grey symbols) donors were incubated with GM-CSF (100 pg/mL), IFN-*γ* (10 ng/mL), or both for the indicated time periods and analysed by flow cytometry. Lines show median values, *p < 0.05, Wilcoxon signed-rank test.

In a first approach, we studied whether GM-CSF, IFN-*γ*, or their combination affect the expression of CD16, CD32, and CD64 on neutrophils after 24, 48, and 72 h. In addition, the percentage of HLA-DR^+^ neutrophils was determined. Neutrophils from three allergic and three non-allergic individuals were analysed and revealed comparable results (Figure 1). Overall, IFN-*γ* and its combination with GM-CSF induced a significant upregulation of Fc*γ*RI/CD64 whereas GM-CSF alone showed this effect only after 72 h. The constitutive expression of Fc*γ*RII/CD32A/B remained unchanged in response to GM-CSF and both cytokines and decreased slightly over time in the presence of IFN-*γ*. Neutrophils from neither allergic nor non-allergic donors expressed CD32B/C (data not shown). Furthermore, each cytokine and their combination significantly reduced Fc*γ*RIII/CD16 expression over time. The addition of IL-3 to GM-CSF and IFN-*γ* had no effect on the Fc*γ*R expression profile (data not shown). In alignment with previous results, GM-CSF, IFN-*γ* and their combination significantly enhanced the percentage of HLA-DR-positive neutrophils (14).

### Formation of allergen-antibody immune complexes

**Figure 2:**
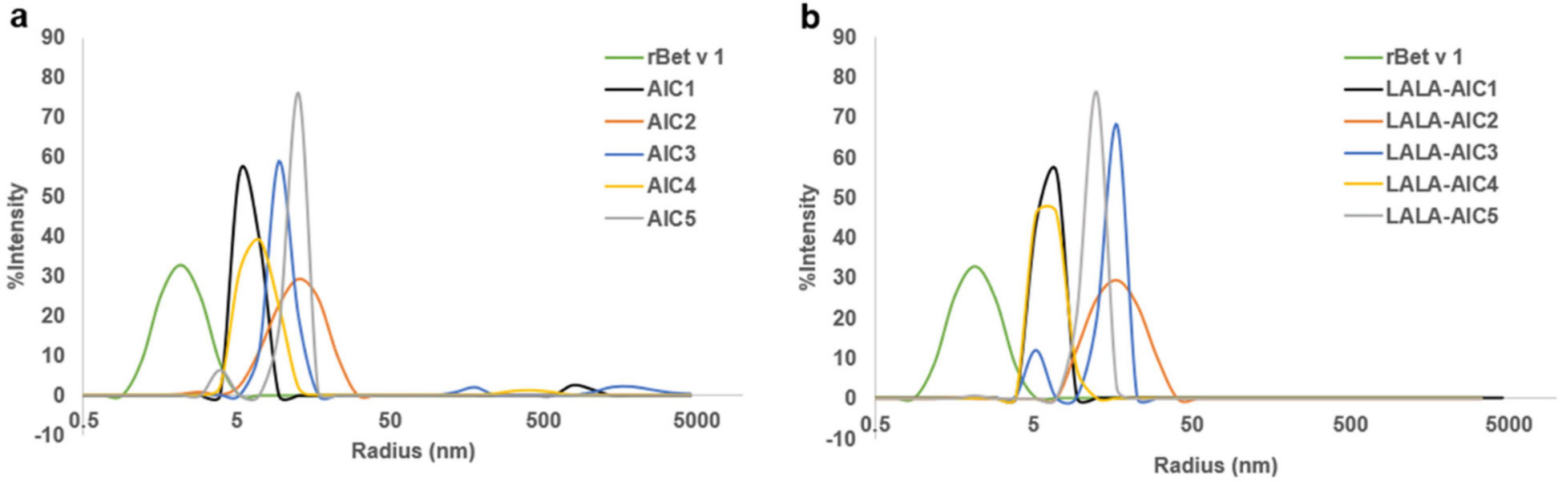
Detection of AIC by DLS. Hydrodynamic radius distribution by intensity for rBet v 1, rBet v 1 after incubation with mAb1 (AIC1), with two mAbs (AIC2, AIC3, AIC4) and three mAbs (AIC5). **(a)** native mAbs; **(b)** LALA variants. Data show complete mean DLS profiles from 20 records per sample.

rBet v 1 was incubated with equimolar concentrations of three mAbs or their LALA variants in different combinations. Figure 2a shows the hydrodynamic radius distribution by intensity for rBet v 1, rBet v 1 plus mAb1 (AIC1), rBet v 1 plus mAb1 and mAb2 (AIC2), rBet v 1 plus mAb 1 and mAb3 (AIC3), rBet v 1 plus mAb2 and mAb3 (AIC4), and rBet v 1 plus mAb1, mAb2, and mAb3 (AIC5). For each sample, a major peak was detected. Small peaks between 50 nm and 5,000 nm appeared in AIC1, AIC3, and AIC4, suggesting the presence of high molecular weight aggregates. Still, their amounts were negligible. Compared to AIC1, a shift towards larger particles was observed for AIC2, AIC3, and AIC5, but not for AIC4. Moreover, the peak of AIC5 overlapped with those of AIC2 and AIC3, indicating that incubation of rBet v 1 with all three mAbs failed to form larger complexes than obtained with two mAbs. Similar complex sizes were obtained when rBet v 1 was incubated with the LALA variants of the mAbs (Figure 2b). Together, these observations indicated that mAb2 and mAb3 hindered each other to simultaneously bind to rBet v 1. As a consequence, we did not use AIC4 and AIC5 and their LALA variants in further experiments.

### Uptake of non-complexed and complexed allergen by neutrophils

**Figure 3:**
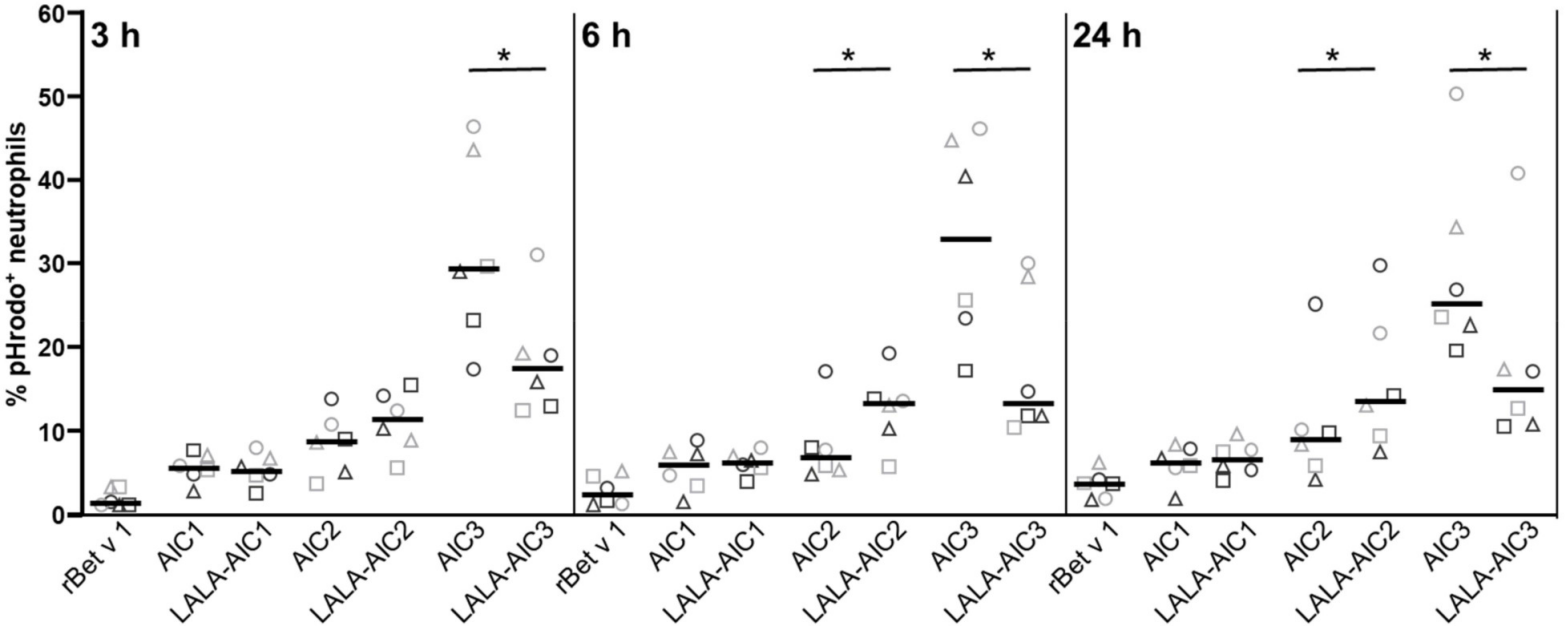
Phagocytosis of non-complexed and IgG1-complexed allergen by neutrophils. Neutrophils from 3 non-allergic (bright grey symbols) and 3 birch pollen-allergic individuals (dark grey symbols) were incubated with GM-CSF/IFN-*γ*/IL-3 and pHrodo-labeled rBet v 1 or the indicated allergen-antibody complexes. The percentage of pHrodo^+^ cells was assessed by using flow cytometry at the indicated time points. Lines show median values, *p < 0.05, Wilcoxon signed-rank test.

To study phagocytosis we employed rBet v 1 labeled with pHrodo™ Red dye that emits fluorescence when the pH value is reduced by fusion of phagosomal and granular vesicles. Neutrophils were incubated with pHrodo-labelled rBet v 1 or its complexes with mAbs and their LALA variants for 3, 6, and 24 h, respectively. No differences were detected between allergic and non-allergic individuals (Figure 3). Summarizing all donors, we observed that the proportion of pHrodo^+^ neutrophils incubated with rBet v 1 increased continuously (3 h–6 h *P* = 0.028 and 3 h–24 h *P* = 0.028, Wilcoxon Signed Ranks test) (Figure 3). In contrast, the proportion of pHrodo^+^ neutrophils incubated with the immune complexes remained at levels recorded after 3 h. At any point in time, incubation with complexed allergen resulted in significantly higher numbers of pHrodo^+^ cells than incubation with non-complexed allergen (for all *P* = 0.028). The internalisation of AIC1 and LALA-AIC1 was consistently equal. After 6 and 24 h, the uptake of AIC2 was significantly lower than of LALA-AIC2. AIC3 was internalised more effectively than LALA-AIC3 at every incubation period.

### Induction of neutrophil extracellular traps by non-complexed and complexed allergen

**Figure 4:**
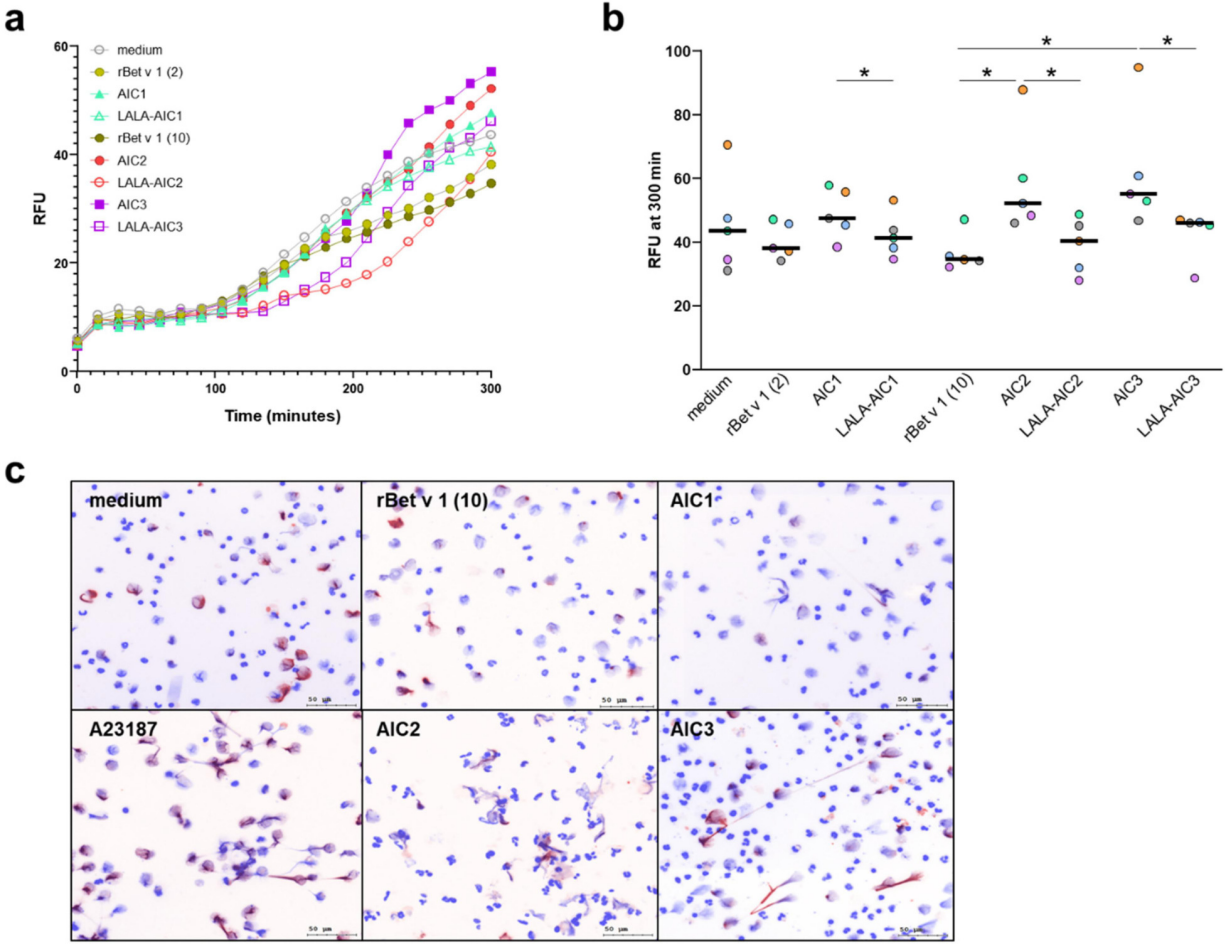
Neutrophil extracellular trap formation in response to allergen or allergen-antibody complexes. Neutrophils from 5 birch-pollen allergic individuals were stimulated with rBet v 1 (2 and 10 µg/mL) and corresponding amounts of immune complexes. Extracellular DNA release was quantified by SYTOX Green fluorescence measured every 15 min and expressed as relative fluorescence units (RFU). **(a)** median values of DNA release of all 5 individuals over time. **(b)** endpoint analysis at 300 min; donors are shown in different colours, the line indicates the median value, *p < 0.05, Wilcoxon signed-rank test. **(c)** NETs were visualized at 180 min by immunofluorescence staining for citrullinated histone H4 (CitH4, red) and DNA (blue); representative overlay images are shown on a white background to facilitate visualization of DNA strands, scale bar: 50 μm.

Neutrophils from 5 birch pollen-allergic donors were incubated with rBet v 1, AICs or LALA-AICs and extracellular DNA was assessed by incorporation of Sytox Green dye and measurement of RFU over 5 h (Figure 4a). Comparison of DNA release at the endpoint (300 min) showed weak responses to AIC1 and stronger responses to AIC2 and AIC3 (Figure 4b). In all cases, AIC-induced DNA release was significantly stronger than the respective LALA-variants. Non-complexed rBet v 1 did not induce NET formation. The findings were further confirmed by immunofluorescence microscopy of NET formation, with representative images from one donor shown for rBet v 1, AIC1, AIC2, and AIC3 (Figure 4c).

### Antigen-presentation of neutrophils primed with non-complexed and complexed allergen

**Figure 5:**
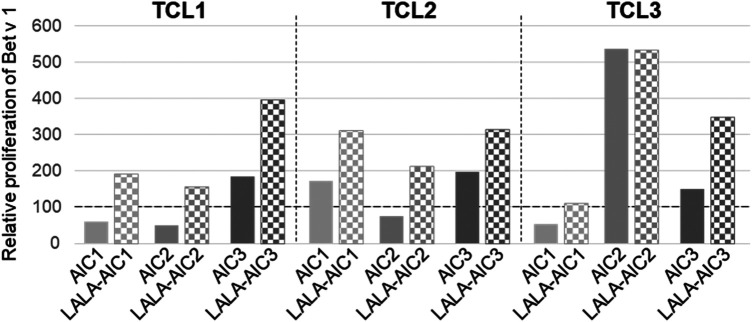
T cell response to complexed and non-complexed allergen presented by neutrophils. Neutrophils were incubated with GM-CSF/IFN-*γ*/IL-3, rBet v 1, AICs or LALA-AICs for 24 h, washed once, and added to autologous Bet v 1-specific T cells from 3 different birch-pollen allergic individuals. T cell proliferation was assessed (dpm). Data are presented as relative proliferation of rBet v 1-induced proliferation set to 100%.

Isolated neutrophils were incubated with rBet v 1 or the different AICs and LALA-AICs in the presence of GM-CSF/IFN-*γ*/IL-3 for 24 h, washed once, and added to autologous Bet v 1-specific T cell cultures from three different birch pollen-allergic individuals. T cell proliferation served as read-out for antigen-presentation. rBet v 1-induced proliferation was normalised to 100%. Figure 5 depicts proliferative responses to AICs and LALA-AICs in relation to proliferation triggered by rBet v 1. T cell proliferation in response to AIC1 and AIC2 was lower than to non-complexed rBet v 1 in 2/3 T cell cultures. In contrast, neutrophils that had been primed with AIC3 and LALA-AICs in particular induced more pronounced T cell proliferation than those incubated with non-complexed allergen in all T cell cultures.

## Discussion

Regular natural exposure to or therapeutic administration of allergens prompts high levels of allergen-specific IgG antibodies. The induction of high affinity IgG antibodies that simultaneously bind to allergens and thereby cover their entire surface, is desired in AIT as it may prevent allergen binding of IgE and ameliorates the classical pathway involving IgE/mast cell (basophil)/histamine. In this regard, cocktails of two and three mAbs specific for the major allergens in cat and birch pollen, respectively, were developed for passive immunotherapy of allergy (24, 25). A single administration of these cocktails resulted in a clinically meaningful reduction of symptoms over a period of 85 days in nasal provocation tests performed every three to four weeks. In parallel, the reactivity in skin prick tests was reduced for up to 113 days after injection (35). Together, these findings indicated that circulating mAbs form AICs with inhaled allergen that prevented IgE-mediated reactions in the respiratory tract and the skin. Immune complexes bind to Fc*γ*R expressed on neutrophils and induce their activation resulting in phagocytosis and the production of ROS, NETs, and cytokines (36). This knowledge tempted us to investigate whether neutrophils also respond to allergen-antibody complexes. We employed three different rBet v 1-specific mAbs to produce AICs *in vitro*. Two of these mAbs were derived from birch pollen-allergic individuals who had received daily sublingual doses of rBet v 1 for 16 weeks (29). Despite being established from different persons, the mAbs seemed to recognize overlapping epitopes, thus, preventing their simultaneous binding to the allergen. Therefore, our largest AICs consisted of rBet v 1 and two mAbs.

To transform neutrophils into APC, they require priming with GM-CSF and/or IFN-*γ* and/or IL-3 to trigger HLA-DR expression on their surface (14). Exposure to these cytokines also affected their Fc*γ*R expression: While CD32 was not altered, CD16 was downregulated and CD64 was upregulated over time with no differences between neutrophils from allergic and non-allergic individuals. Antigen uptake is another important feature of APC and CD32 is the major receptor for Fc*γ*R-mediated phagocytosis of neutrophils (4, 37). Thus, we were interested to see whether neutrophils internalise AICs more efficiently than non-complexed allergen by involving this pathway. Neutrophils engulfed AICs more rapidly and effectively than non-complexed rBet v 1. Phagocytosis of AICs was influenced by their size with complexes containing two mAbs being more effectively internalised than complexes containing one mAb. these findings point to particle uptake-driven phagocytosis of allergen-antibody complexes (38). Since internalization of both complexes containing two mAbs, AIC2 and AIC3, showed opposing trends with respect to the involvement of Fc*γ*R we examined potential differences between the two. AIC2 contained mAb2 which has a lower affinity for Bet v 1 than mAb3 contained in AIC3. Furthermore, DLS measurements showed a larger heterogeneity of AIC2 than AIC3 as indicated by polydispersity indexes of 0.1 and 0.03, respectively. Taken together, these results demonstrated that AIC3 is more stable than AIC2. Consequently, we considered AIC3 as a more reliable tool for inferring the involvement of Fc*γ*R and conclude that Fc*γ*R-mediated phagocytosis further enhances the uptake of allergen-antibody complexes by neutrophils.

To assess whether enhanced allergen uptake through phagocytosis of AICs induces stronger T cell responses, we co-cultured allergen-specific T cells with autologous cytokine-primed neutrophils together with rBet v 1, AICs, or LALA-AICs. T cell proliferation was consistently increased when neutrophils had been exposed to LALA-AICs but varied greatly in response to AICs. Given that the aggregation of CD16 by immune complexes triggers NET release (39–41) we subjected AIC-stimulated neutrophils from allergic donors to DNA-release assays. While AICs stimulated NET release to a varying degree, LALA-AICs failed in NET induction. Considering these results it is tempting to speculate that AIC-triggered NET formation and associated T cell suppression may—at least in part—explain the discrepant outcome of T cell activation and phagocytosis assays. NETs have been shown to suppress T cell responses (42–44). In addition to DNA, NETs also contain histones and proteins from azurophilic (primary), specific (secondary), as well as tertiary granules which may be toxic to T cells. Another aspect to consider is that the NET release process may induce cell death (NETosis) (45), thereby decreasing the numbers of allergen-pulsed neutrophils serving as APC for T cells. Indeed, we observed significantly higher percentages of dead neutrophils after incubation with AICs than with LALA-AICs (data not shown). Thus, Fc*γ*R-mediated NET induction by AICs may interfere with the T cell stimulatory properties of neutrophils and their APC function after allergen/AIC phagocytosis. However, our *in vitro* results cannot be readily extrapolated to the *in vivo* situation. While all released products accumulate and act simultaneously in co-cultures, the observed activation processes may occur with a time lag in the tissue and could be attenuated by the local environment. Therefore, no conclusions can currently be drawn about the allergen-presenting potency of neutrophils that encounter AICs *in vivo*.

Nevertheless, to the best of our knowledge, this study is the first to describe neutrophilic responses to IgG1 complexes with a major respiratory allergen of clinical relevance. Our results suggest a novel link between humoral and cellular allergen-specific immune responses.

## Data Availability

The raw data supporting the conclusions of this article will be made available by the authors, without undue reservation.
